# Secretion and Delivery of Intestinal Pathogenic *Escherichia coli* Virulence Factors via Outer Membrane Vesicles

**DOI:** 10.3389/fcimb.2020.00091

**Published:** 2020-03-06

**Authors:** Christian Rueter, Martina Bielaszewska

**Affiliations:** ^1^Center for Molecular Biology of Inflammation (ZMBE), Institute of Infectiology, University of Muenster, Münster, Germany; ^2^National Institute of Public Health, Reference Laboratory for E. coli and Shigellae, Prague, Czechia; ^3^Institute for Hygiene, University Hospital of Muenster, University of Muenster, Münster, Germany

**Keywords:** outer membrane vesicles, intestinal pathogenic *Escherichia coli*, EHEC, ETEC, virulence factors, toxins

## Abstract

Outer membrane vesicles (OMVs) are nanoscale proteoliposomes secreted from the cell envelope of all Gram-negative bacteria. Originally considered as an artifact of the cell wall, OMVs are now recognized as a general secretion system, which serves to improve the fitness of bacteria and facilitate bacterial interactions in polymicrobial communities as well as interactions between the microbe and the host. In general, OMVs are released in increased amounts from pathogenic bacteria and have been found to harbor much of the contents of the parental bacterium. They mainly encompass components of the outer membrane and the periplasm including various virulence factors such as toxins, adhesins, and immunomodulatory molecules. Numerous studies have clearly shown that the delivery of toxins and other virulence factors via OMVs essentially influences their interactions with host cells. Here, we review the OMV-mediated intracellular deployment of toxins and other virulence factors with a special focus on intestinal pathogenic *Escherichia coli*. Especially, OMVs ubiquitously produced and secreted by enterohemorrhagic *E. coli* (EHEC) appear as a highly advanced mechanism for secretion and simultaneous, coordinated and direct delivery of bacterial virulence factors into host cells. OMV-associated virulence factors are not only stabilized by the association with OMVs, but can also often target previously unknown target structures and perform novel activities. The toxins are released by OMVs in their active forms and are transported via cell sorting processes to their specific cell compartments, where they can develop their detrimental effects. OMVs can be considered as bacterial “long distance weapons” that attack host tissues and help bacterial pathogens to establish the colonization of their biological niche(s), impair host cell function, and modulate the defense of the host. Thus, OMVs contribute significantly to the virulence of the pathogenic bacteria.

## Introduction

Outer membrane vesicles (OMVs) are nanoscale proteoliposomes secreted from the cell envelope of all Gram-negative bacteria (Amano et al., 2010; Ellis and Kuehn, 2010; Kulp and Kuehn, 2010; O'Donoghue and Krachler, 2016). They are produced by a controlled blebbing of the bacterial outer membrane due to the envelope disturbances via different mechanisms (Kulp and Kuehn, 2010; Schwechheimer and Kuehn, 2015; Elhenawy et al., 2016; Roier et al., 2016; Toyofuku et al., 2019). As a result, OMVs are surrounded by a single membrane bilayer and contain mostly components of the bacterial outer membrane (outer membrane proteins, lipopolysaccharide, phospholipids, peptidoglycan) and the periplasm (periplasmic proteins) (Kulp and Kuehn, 2010; Schwechheimer and Kuehn, 2015; Toyofuku et al., 2019). Originally considered an artifact of the cell wall, OMVs are now recognized as a general secretion system (Guerrero-Mandujano et al., 2017), which serves to improve the fitness of bacteria and facilitate interactions between cells in the context of mixed bacterial communities and between host and microbe (Ellis and Kuehn, 2010; Kulp and Kuehn, 2010; MacDonald and Kuehn, 2012; Haurat et al., 2015). The release of membrane vesicles is an ubiquitous process and was observed among a wide range of bacteria. Not only pathogenic bacteria such as for example *Vibrio cholerae, Campylobacter jejuni, Helicobacter pylori, Aggregatibacter actinomycetemcomitans, Pseudomonas aeruginosa, Moraxella catarrhalis, Stenotrophomonas maltophilia, Acinetobacter baumannii, Shigella flexneri, Salmonella enterica* serovar Typhimurium, enterotoxigenic *Escherichia coli* (ETEC), enterohemorrhagic *E. coli* (EHEC), adherent-invasive *E. coli*, and extraintestinal pathogenic *E. coli*, but also non-pathogenic bacteria such as *E. coli* Nissle 1917, shed membrane vesicles during growth (Kadurugamuwa and Beveridge, 1995, 1997; Wai et al., 1995, 2003; Horstman and Kuehn, 2000, 2002; Kesty et al., 2004; Balsalobre et al., 2006; Kouokam et al., 2006; Bomberger et al., 2009; Lindmark et al., 2009; Ellis and Kuehn, 2010; Rolhion et al., 2010; Chatterjee and Chaudhuri, 2011; Rumbo et al., 2011; Schaar et al., 2011; Rompikuntal et al., 2012, 2015; Guidi et al., 2013; Kunsmann et al., 2015; Elhenawy et al., 2016; Bielaszewska et al., 2017; Chatterjee et al., 2017; Devos et al., 2017; Fabrega et al., 2017; Svennerholm et al., 2017; Wang et al., 2019).

**Figure 1:**
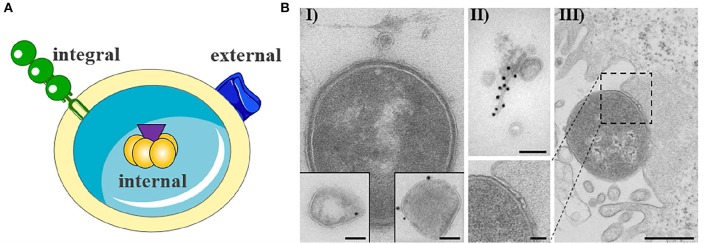
Outer membrane vesicle from gram negative bacteria. **(A)** Schematic representation of OMV with cargo. The cargo may either be present as a solute in the vesicle lumen (internal) or be associated with (external) or integrated into the vesicle membrane (integral). The figure was produced using Servier Medical Art. **(B)** Exemplary electron microscopy pictures of outer membrane vesicles (based on Bielaszewska et al., 2013, 2017). (I) OMV released from the entero-hemorrhaghic *E. coli*. Virulence factors can either be internal (immunogold labeling of Stx2a, left inset) or associated (immunogold labeling of EHEC-Hly, right inset) with OMVs (scale bar 100 nm). (II) OMVs are often associated with flagelin (immunogold labeling of flagellin, scale bar 100 nm). (III) Bacteria are also able to release OMVs to host cells during infection (scale bar 500 nm).

OMVs typically have a diameter of 20–250 nm and are released during all growth phases and under all environmental conditions (Ellis and Kuehn, 2010; Bonnington and Kuehn, 2014). OMVs protect their molecular biological content against the external environment and can transport their cargo over long distances (Bomberger et al., 2009; Bonnington and Kuehn, 2014). The cargo may either be present as a solute in the vesicle lumen or be associated with or integrated into the vesicle membrane (Horstman and Kuehn, 2000; Kesty et al., 2004; Bomberger et al., 2009; Lindmark et al., 2009; Bielaszewska et al., 2013, 2017; Kunsmann et al., 2015; Figures 1A,BI,II). OMVs carry both bacterial toxins (Horstman and Kuehn, 2000; Wai et al., 2003; Kesty et al., 2004; Balsalobre et al., 2006; Kouokam et al., 2006; Aldick et al., 2009; Ellis and Kuehn, 2010; Chatterjee and Chaudhuri, 2011; Rompikuntal et al., 2012; Guidi et al., 2013; Kunsmann et al., 2015; Bielaszewska et al., 2017) and other virulence factors such as adhesins, invasins, outer membrane proteins, lipopolysaccharide (LPS), flagellin, and proteases (Kadurugamuwa and Beveridge, 1995; Bomberger et al., 2009; Ellis and Kuehn, 2010; Rolhion et al., 2010; Kunsmann et al., 2015; Rompikuntal et al., 2015; Vanaja et al., 2016; Bielaszewska et al., 2017). Secretion of OMVs is generally considered to be an adaptive response to environmental stress and often occurs during infection when the bacteria are exposed to the host's defense mechanisms (MacDonald and Kuehn, 2012; Orench-Rivera and Kuehn, 2016; Bauwens et al., 2017b). In the presence of antimicrobial peptides or bacteriophages, increased production of membrane vesicles correlates with improved fitness and increased survival (Manning and Kuehn, 2011; Duperthuy et al., 2013). For example, EHEC enhances the secretion of outer membrane protease OmpT-loaded OMVs during infection and thereby blocks bacterial cell attack by human antibacterial peptide cathelicidin LL-37 (Urashima et al., 2017).

In general, OMVs are released in increased amounts from pathogenic bacteria, suggesting that OMV secretion is an additional virulence mechanism of pathogens (Horstman and Kuehn, 2000; Ellis and Kuehn, 2010). OMVs can take on both defensive and offensive tasks during infection (MacDonald and Kuehn, 2012). Defensively, they can be used to sequester antibiotics, bacteriophages, and antibodies, bind or degrade antimicrobial peptides, as well as bait antigens to distract the immune system (Manning and Kuehn, 2011; MacDonald and Kuehn, 2012; Duperthuy et al., 2013; O'Donoghue and Krachler, 2016; Urashima et al., 2017; Reyes-Robles et al., 2018). The potential of OMVs as offensive weapons is evident in their ability to deliver virulence factors into host cells (Figure 1BIII) (Kesty et al., 2004; Tan et al., 2007; Bomberger et al., 2009; Amano et al., 2010; Ellis and Kuehn, 2010; Kulp and Kuehn, 2010; Schaar et al., 2011; Rompikuntal et al., 2012; Bielaszewska et al., 2013, 2017; Kunsmann et al., 2015; O'Donoghue and Krachler, 2016; Rüter et al., 2018) as well as to induce sepsis, sepsis-associated cardiomyopathy or disseminated intravascular coagulation in the absence of intact bacterial cells (Park et al., 2010; Shah et al., 2012; Svennerholm et al., 2017; Wang et al., 2019). The OMV-associated LPS does not only appear to be effective through the extracellular Toll-like receptor (TLR) 4 (Kunsmann et al., 2015; Bielaszewska et al., 2018; Wang et al., 2019) or TLR2 (Schaar et al., 2011), as OMV-bound LPS can also activate the non-canonical inflammasome signaling pathway intracellularly after uptake of the OMVs into the target cells (Vanaja et al., 2016). The uptake of OMV occurs either by phagocytosis or by classic endocytosis (Kesty et al., 2004; Bielaszewska et al., 2013, 2017; Rewatkar et al., 2015; O'Donoghue and Krachler, 2016), whereupon the virulence factors differentially separate during the intracellular transport of the OMVs and can develop their toxic activities (Bielaszewska et al., 2017). Many “well-known” virulence factors and toxins have been identified that use OMVs as an alternative secretory pathway. However, some toxins, such as EHEC cytolysin ClyA, EHEC cytolethal distending toxin V, ETEC heat-labile enterotoxin (LT), *Shigella* enterotoxin 1 (ShET1), and *C. jejuni* cytolethal distending toxin, seem to use OMVs exclusively as a secretory pathway (Horstman and Kuehn, 2000, 2002; Wai et al., 2003; Kesty et al., 2004; Lindmark et al., 2009; Kunsmann et al., 2015; O'Donoghue and Krachler, 2016; Bielaszewska et al., 2017). Beyond their'canonical' functions, the packaging of virulence factors into or onto OMVs concentrates and increases the stability of virulence factors, allows a differential intracellular release of toxins and other virulence factors, targets specific virulence factors to distinct organelles in the host cell, which can broaden or change their functions, and allows their transport for long distances (Aldick et al., 2009; Bomberger et al., 2009; Kulp and Kuehn, 2010; Bielaszewska et al., 2013, 2017; Vanaja et al., 2016; Rüter et al., 2018).

## Biogenesis of OMVs

Different mechanisms for OMV biogenesis have been described so far. One mechanism proposes the temporary reduction or relocation of covalent linkages of proteins between the outer membrane (OM) and the peptidoglycan (PG). At the site of local decrease in overall crosslinks, the OM has to grow fast and finally bud off (Schwechheimer and Kuehn, 2015). This model was supported by a study in which mutants lacking OmpA and thus harboring a lower number of crosslinks between OM and PG, revealed an increased OMV production (Kulp and Kuehn, 2010). Another model of OMV biogenesis includes the accumulation of misfolded proteins or envelope components such as PG fragments in the periplasm (Schwechheimer and Kuehn, 2015). Local assembly of these components might be able to induce a periplasmic turgor pressure on the OM. As a consequence, the OM protrudes, encapsulates undesirable components, and pinches off. The accumulation of proteins or envelope components at a specific area in the periplasm might be further attracted by a depletion of covalent crosslinks between the OM and PG (Kulp and Kuehn, 2010). Another mechanism of OMV biogenesis is based on a species- specific *P. aeruginosa* model. The signaling molecule Pseudomonas quinolone signal (PQS) was shown to influence and modify the membrane curvature (Roier et al., 2016). PQS can be inserted into the OM and is able to bind to LPS. This leads to the loss of linkages between the OM and PG and induces periplasmic turgor pressure. Due to binding of positively charged components and reduction of Mg^2+^ and Ca^2+^ salt bridges, anionic repulsions between LPS molecules increase causing the OM to pinch off (Schwechheimer and Kuehn, 2015; Jan, 2017). Another promising mechanism of OMV biogenesis is more general and might be conserved among different bacterial species. The model includes a regulated ABC transport system for phospholipids which is spanned from the OM to the cytoplasmic membrane (Roier et al., 2016). The gene cluster *yrb* (*yrbB*-*yrbE*) as well as the lipoprotein VacJ have been shown to be part of the system. Maintaining the lipid asymmetry in the OM, it regulates the retrograde transport from the OM to the inner membrane avoiding accumulation of phospholipids. Roier et al. (2016) showed by deletion or reduced expression of the transporter that the OMV production was increased and phospholipids assembled close to the OM. Moreover, under iron-limited conditions the transport system was downregulated dependent on the ferric uptake regulator (Fur) suggesting that increased OMV production is regulated indirectly by Fur and the phospholipid transporter (Roier et al., 2016).

## Increased OMV Production as a Bacterial Stress Response

Larger amounts of membrane vesicle release has been broadly observed related to stress response of bacteria. Nutrient scarcity, iron limitation, oxidative stress, hydrogen peroxide as well as a low pH induced the release of OMVs in high amounts (Schwechheimer and Kuehn, 2015; Orench-Rivera and Kuehn, 2016; Bauwens et al., 2017b). OMVs seem to provide resistance and dispose defensive mechanisms against environmental stressors (MacDonald and Kuehn, 2012; Orench-Rivera and Kuehn, 2016). Furthermore, OMVs seem to facilitate adaptation to a challenging environment and increase the chance of bacterial survival (Duperthuy et al., 2013; Bauwens et al., 2017b; Urashima et al., 2017). Stressors can be of physical, chemical or biological nature. Temperature as a stressor has often been described. Higher temperature can lead to denaturation or misfolding of proteins which results in accumulation in the periplasmic space (Guerrero-Mandujano et al., 2017). The outer membrane further becomes more fluid by higher temperature which promotes protrusion and OMV production (Kulp and Kuehn, 2010). In contrast, a reduction in temperature leads to an increased OMV release in the cold-adapted bacterium *Shewanella livingstonensis*, the soil bacterium *Serratia marcescens* and the pathogen *Bartonella henselae* (Schwechheimer and Kuehn, 2015). Increased vesicle production can also occur after treatment with antimicrobials such as the ciprofloxacin, polymyxin B, gentamicin, or beta-lactam antibiotics (Kadurugamuwa and Beveridge, 1995, 1997; Manning and Kuehn, 2011; MacDonald and Kuehn, 2012; Maredia et al., 2012; Bauwens et al., 2017a; Devos et al., 2017). Interestingly, on the one hand, vesicles are able to carry resistance determinants, either a resistance gene or a respective degradative enzyme, to prevent damage of the bacterial cell (Ciofu et al., 2000; Rumbo et al., 2011; Fulsundar et al., 2014; Schaar et al., 2014; Chattopadhyay and Jagannadham, 2015; Stentz et al., 2015; Devos et al., 2016; González et al., 2016; Chatterjee et al., 2017; Domingues and Nielsen, 2017; Kim et al., 2018). On the other hand, OMV-mediated absorption and subsequent inactivation or degradation of antimicrobials of the surrounding environment has been demonstrated (Manning and Kuehn, 2011; Duperthuy et al., 2013; Orench-Rivera and Kuehn, 2016; Guerrero-Mandujano et al., 2017; Urashima et al., 2017). Last-mentioned, vesicle production can be increased after bacteriophage infection. By exposure of receptors on the vesicle surface, the T4-phage was bound and inactivated (Manning and Kuehn, 2011). Phage-particles were encapsulated and this prevented damage of the bacterial cell (Manning and Kuehn, 2011; Guerrero-Mandujano et al., 2017). Increased OMV production provides many advantages against environmental stressors not only *in vitro* but also in the human host as demonstrated by significantly upregulated vesiculation in EHEC under simulated human intestinal conditions and in the human intestine (Bauwens et al., 2017b).

It is worth mentioning that membrane vesicles produced under particular environmental stress may not result from the outer membrane blebbing typical for the formation of OMVs, but arise by different mechanisms and thus differ from OMVs by their composition and presumably by their functions. Specifically, the outer-inner membrane vesicles (OIMVs) recently identified in several genera of Gram-negative bacteria including pathogens such as *Neisseria gonorrhea, Pseudomonas aeruginosa*, and *Acinetobacter baumannii* (Pérez-Cruz et al., 2013, 2015) are characterized by a double membrane bilayer derived from the outer and the inner membrane, respectively. Due to their origin, OIMVs carry, besides membrane components, also cytoplasmic components including DNA (Pérez-Cruz et al., 2015; Toyofuku et al., 2019) and has been thus proposed as major vesicles type involved in the DNA transfer (Toyofuku et al., 2019). OIMVs have been suggested to result from an explosive cell lysis triggered by a phage-derived endolysin that degrades the cell wall peptidoglycan (Turnbull et al., 2016); by reassembling of fragments of the outer and inner membrane of the lysed cells OIMVs arise, whereas reassembling of outer membrane fragments gives rise to so called explosive OMVs (EOMVs) (Turnbull et al., 2016; Toyofuku et al., 2019). The observation of OIMVs and EOMVs formation after bacterial treatment with SOS response-triggering agents such as ciprofloxacin and mitomycin C (Turnbull et al., 2016; Devos et al., 2017) which also induce temperate bacteriophages including those encoding endolysin (Turnbull et al., 2016; Devos et al., 2017) suggests that formation of these vesicles results from phage-mediated cell lysis triggered by the SOS response (Toyofuku et al., 2019).

## OMVs as an Alternative Secretion System: Type-0 Secretion System

Besides the well-established secretion-systems 1-6, OMVs have been recently considered as a new independent type-0 secretion system (T0SS) (Guerrero-Mandujano et al., 2017). OMVs not only secrete misfolded proteins or toxic products as described above, but they are also able to transport different types of macromolecules. Because of the lipophilic structure of OMVs, secretion of lipids, hydrophobic and insoluble proteins is facilitated (Guerrero-Mandujano et al., 2017). Moreover, the bilayered envelope of OMVs provides protection against physical and chemical influences as well as enzymatic degradation. OMVs provide unique advantages against other secretion systems by transporting proteins in high concentrations and delivering them to target destinations over long distances (Bomberger et al., 2009; Kulp and Kuehn, 2010; Guerrero-Mandujano et al., 2017). Many studies have reported that the cargo of OMVs is selectively packaged and certain molecules are enriched or excluded (Kesty and Kuehn, 2004; Schwechheimer and Kuehn, 2015). The delivery of bacterial effector proteins by OMVs into host cells seems to be a crucial aspect for pathogens (Figure 1BIII). In this regard, several investigations have demonstrated that budded portions of outer membrane material are shed also *in vivo*: vesicles produced by *H. pylori* were found in human gastric epithelium biopsies (Fiocca et al., 1999), and outer membrane protein–LPS complexes have been found in the sera of patients and rats with sepsis caused by *Enterobacteriaceae* (Hellman et al., 2000), in the plasma and the cerebrospinal fluid of patients with N*eisseria meningitidis* sepsis and meningitis, respectively (Stephens et al., 1982; Namork and Brandtzaeg, 2002), and in the nasal mucosa of a patient with sinusitis caused by *M. catarrhalis* (Tan et al., 2007). Vesicles from pathogenic strains such as *Pseudomonas aeruginosa, H. pylori, A. actinomycetemcomitans, C. jejuni, S. enterica, V. cholera*, and pathogenic *E. coli* contain active virulence factors, such as proteases, pro-inflammatory proteins, LPS, and toxins (Kadurugamuwa and Beveridge, 1995, 1997; Kolling and Matthews, 1999; Horstman and Kuehn, 2000; Keenan and Allardyce, 2000; Kato et al., 2002; Wai et al., 2003; Kesty et al., 2004; Kouokam et al., 2006; Lindmark et al., 2009; Ellis and Kuehn, 2010; Kaparakis et al., 2010; Chatterjee and Chaudhuri, 2011; Schaar et al., 2011; Rompikuntal et al., 2012, 2015; Bielaszewska et al., 2013, 2017, 2018; Guidi et al., 2013; Elluri et al., 2014; Thay et al., 2014; Kunsmann et al., 2015). However, the molecular mechanism of virulence factor delivery via vesicles has been unclear. In addition to their production during infection, the key role of OMVs in bacterial virulence is supported by their ability to mimic in animal models diseases caused by the parental pathogens (Kim et al., 2011; Shah et al., 2012; Svennerholm et al., 2017), and to induce protective immune responses (Roy et al., 2011; Leitner et al., 2015; Roier et al., 2016; Liu et al., 2017).

## OMV-Associated Toxins and Other Virulence Factors From Intestinal Pathogenic *Escherichia coli*

Intestinal pathogenic *E. coli* such as ETEC and EHEC produce OMVs under laboratory conditions as well as during infection (Wai et al., 1995; Kolling and Matthews, 1999; Horstman and Kuehn, 2000; Yokoyama et al., 2000; Kesty et al., 2004; Aldick et al., 2009; Ellis and Kuehn, 2010; Bielaszewska et al., 2013, 2017; Kunsmann et al., 2015; Bauwens et al., 2017b). Vesicles may contribute to the bacterial pathogenicity by serving as vehicles for toxin delivery into host cells (Kesty et al., 2004; Bielaszewska et al., 2013, 2017) as well as by inducing an inflammatory response, in particular secretion of interleukin 8 (IL-8) from intestinal epithelial cells (Kunsmann et al., 2015; Bielaszewska et al., 2018). Most vesicle proteins were resistant to dissociation, suggesting they were integral, or internal (Bielaszewska et al., 2013, 2017). In some cases, virulence factors can also be tightly attached to the vesicle surface (Bielaszewska et al., 2013; Kunsmann et al., 2015). Table 1 shows an overview of virulence factors associated with OMVs from pathogenic *E. coli*, which will be discussed in the following text separately for the OMV-mediated delivery of toxins and other virulence factors from ETEC and EHEC.

**Table 1:** Overview of virulence factors associated with OMVs from intestinal pathogenic *E. coli*.

**Type of pathogenic *E. coli***	**OMV associated virulence factor**	**Function**	**Association with OMV**	**Vesicle formation**	**References**
Enterotoxigenic *E. coli*	Heat labile enterotoxin (LT)	Cytotoxicity	Internal and external	Blebbing	Horstman and Kuehn, 2000, 2002
(ETEC)					Kesty et al., 2004
	EtpA	T-Cell stimulating Protein/Adhesin	Not determined	Not determined	Roy et al., 2010
	CexE	Adhesin	Not determined	Not determined	Roy et al., 2011
	TibA	Autotransport/Adhesin	Not determined	Not determined	Roy et al., 2010
	Flagellin	Not determined	External	Not determined	Roy et al., 2010
Enterohemorrhagic *E. coli*	Shiga toxin 1	Cytotoxicity, Apoptosis	Not determined	Not determined	Yokoyama et al., 2000
(EHEC)	Shiga toxin 2	Cytotoxicity, Apoptosis	Internal	Blebbing	Kolling and Matthews, 1999
	Cytolysin A (ClyA)	Cytotoxicity	Not determined	Blebbing	Wai et al., 2003
	Shigella enterotoxin 1 (ShET1)	Unknown	Internal	Blebbing	Kunsmann et al., 2015
	EHEC hemolysin	Apoptosis	External	Blebbing	Bielaszewska et al., 2013, 2017
	Cytolethal distending toxin V	Cell cycle arrest, apoptosis	Internal	Blebbing	Bielaszewska et al., 2017
	Flagellin	Proinflammatory (IL-8 secretion)	External	Blebbing	Kunsmann et al., 2015
	Outer membrane protease OmpT	Protection against antimicrobial Peptides	Internal	Not determined	Urashima et al., 2017
	Lipopolysaccharide	Proinflammatory (IL-8 secretion)	Integral	Blebbing	Kunsmann et al., 2015

*Information on vesicle formation and association of virulence factors are based on electron microscopy. Formation of vesicles and the proportions of different vesicle types in the culture, even of the same bacterium might differ dependent on culture conditions*.

## OMV-Mediated Delivery of Toxins and Other Virulence Factors From ETEC

ETEC are leading causes of traveler's diarrhea and childhood diarrhea in developing countries (Fleckenstein and Kuhlmann, 2019). The OMV association of the heat-labile enterotoxin (LT), one of the major virulence factors of these strains which disrupts electrolyte balance in the gut epithelium (Mirhoseini et al., 2018), has been demonstrated by several groups (Wai et al., 1995; Horstman and Kuehn, 2000). The toxin is located both inside and on the external of OMVs (Horstman and Kuehn, 2000, 2002) and is biologically active as demonstrated by the ability of LT-carrying OMVs to elicit typical morphological changes on Y1 cells (Horstman and Kuehn, 2000; Kesty et al., 2004). According to the proposed model for LT secretion from ETEC and its interaction with host cells (Horstman and Kuehn, 2002), LT binds, after its secretion from the bacteria via the general secretion pathway, to the bacterial outer membrane via interaction of LT-B subunit with LPS. Subsequently, LT is released from the bacterial cells by budding of OMVs, which contain LT both inside and on the external surface bound to LPS. The external LT binds, via interaction of another site of its B subunit, with GM_1_ cell receptor, tethering thus the vesicle to the host cell (Horstman and Kuehn, 2002). The cellular binding of LT-carrying OMVs to their target cells is thus dependent on the OMV-associated toxin, as has also been reported for OMV-associated cholera toxin of *V. cholerae* (Chatterjee and Chaudhuri, 2011). Cellular binding of LT-carrying OMVs leads to their internalization via lipid rafts and caveolin-dependent endocytosis and internalized vesicles accumulate in a non-acidified compartment of the host cell (Kesty et al., 2004). The pathogenetic role of ETEC OMVs during infection is supported by their increased production *in vivo* (Ellis and Kuehn, 2010) and by their ability to induce immune responses to OMV-associated LT and other virulence proteins such as autotransporter TibA, EtpA adhesin and a novel extracytoplasmic protein CexE (Table 1) (Roy et al., 2010, 2011) which protect against ETEC colonization in a mouse model (Roy et al., 2011; Leitner et al., 2015).

## OMV-Mediated Delivery of Toxins and Other Virulence Factors From EHEC

**Figure 2:**
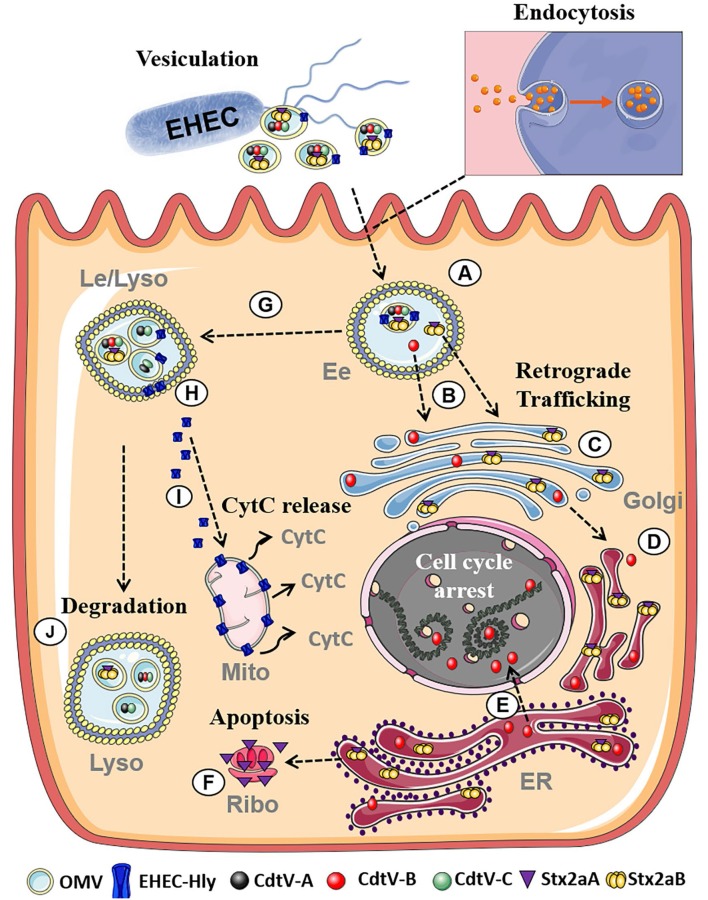
Summary of intracellular trafficking of EHEC O157 OMVs and OMV-delivered toxins (based on Bielaszewska et al., 2013, 2017). After uptake via dynamin-dependent endocytosis, O157 OMVs carrying the toxin cocktail enter the endosomal compartments of target cells **(A)**. Stx2a holotoxin and CdtV-B subunit separate from OMVs in early endosomes **(B)** and are retrogradely transported to the Golgi complex **(C)** and the endoplasmic reticulum **(D)**. From the endoplasmic reticulum, CdtV-B is translocated to the nucleus to target DNA and cause cell-cycle arrest **(E)**, and Stx2a A1 catalytic fragment to the cytosol to reach ribosomes and induce apoptosis **(F)**. CdtV-A and CdtV-C subunits and EHEC-Hly are sorted with OMVs to late endosomes/lysosomes **(G)**. Here EHEC-Hly separates from OMVs, escapes from the lysosomes **(H)**, and is transported to the mitochondria where it causes release of cytochrome C **(I)**. CdtV-A and CdtV-C remain OMV-associated and are degraded with OMVs in lysosomes **(J)**. Moreover, residual subsets of CdtV-B and Stx2a, which did not separate from OMVs in early endosomes, are sorted with OMVs to lysosomes for degradation. Figure was taken from Bielaszewska et al. (2017) and modified using Servier Medical Art. (Ee, Early endosomes; Le, Late endosomes; Lyso, Lysosomes; Golgi, Golgi Apparatus, ER, Endoplasmatic reticulum; Ribo, Ribosome; Mito, Mitochondria, CytC, Cytochrome C).

EHEC are worldwide causes of diarrhea and its severe extraintestinal complication, the hemolytic uremic syndrome (HUS) (Karch et al., 2005; Tarr et al., 2005). EHEC OMVs contain Shiga toxins (Stx) (Table 1; Kolling and Matthews, 1999; Yokoyama et al., 2000; Kunsmann et al., 2015; Bielaszewska et al., 2017), the major EHEC virulence factors involved in the pathogenesis of HUS, which is a thrombotic microangiopathy resulting from Stx-mediated injury of microvascular endothelium, in particular in the renal glomeruli but also in the brain (Zoja et al., 2010; Karpman et al., 2017). Besides Stx, EHEC OMVs also carry other EHEC toxins which injure microvascular endothelium and may thus play roles in the pathogenesis of HUS such as the cytolethal distending toxin V (CdtV) and EHEC hemolysin (EHEC-Hly), as well as flagellin (Aldick et al., 2009; Bielaszewska et al., 2013, 2017; Kunsmann et al., 2015; Table 1). Whereas Stx, EHEC-Hly, and flagellin also exist as free extracellular proteins (Kunsmann et al., 2015; Bielaszewska et al., 2017), CdtV is exclusively secreted via OMVs (Bielaszewska et al., 2017). Studies of interactions of EHEC OMVs and OMV-associated virulence factors with human intestinal epithelial and microvascular endothelial cells, which are the major targets during EHEC infection demonstrated that OMVs with their toxin cargoes are taken up by cells via dynamin-dependent endocytosis (Bielaszewska et al., 2013, 2017) and transported to early and late endosomes (Bielaszewska et al., 2013, 2017) (Figure 2). Here, OMV-associated toxins or their biologically active subunits separate from OMVs and are trafficked, using different pathways, to their cellular targets including ribosomes (Stx2a A subunit), the nucleus (CdtV B subunit), and mitochondria (EHEC-Hly) (Bielaszewska et al., 2013, 2017) (Figure 2). By analyzing biological consequences of OMV-mediated delivery of virulence factors into host cells, we demonstrated that EHEC OMVs that carried Stx2a, CdtV, and EHEC-Hly caused G2 cell cycle arrest followed by apoptosis in human intestinal epithelial and microvascular endothelial cells (Bielaszewska et al., 2013, 2017). CdtV, specifically its B subunit, which possesses the DNase-like activity, was the OMV component responsible for the G2 arrest, whereas all of the OMV-delivered toxins contributed to apoptosis (Bielaszewska et al., 2013, 2017). A detailed analysis of the mechanism of apoptosis caused by OMV-delivered EHEC-Hly demonstrated that after its translocation from late endosomes/lysosomes to the mitochondria, EHEC-Hly causes permeabilization of the inner and outer mitochondrial membranes, which leads to the decrease of the mitochondrial membrane potential, release of cytochrome C from the mitochondria to the cytoplasm, and, as a consequence, activation of caspase-9, the initiator caspase of the intrinsic apoptotic pathway. Subsequent activation of the effector caspase-3 via caspase-9 triggers the apoptotic cell death (Bielaszewska et al., 2013). The failure of OMVs that lacked the EHEC toxins to cause the G2 arrest and apoptosis indicates that the OMV-delivered toxins accounted for the OMV-mediated biological effects (Bielaszewska et al., 2013, 2017). Importantly, the report by Kim et al. (2011) that EHEC O157 OMVs carrying Stx2a, the major EHEC virulence factor, elicited a HUS-like disease in a mouse model supports the hypothesis that OMV-mediated secretion and delivery of EHEC toxins into host cells *in vivo* essentially contribute to the pathogenesis of EHEC infection.

Besides their endothelial cytotoxicity, EHEC OMVs induce secretion of IL-8 from human intestinal epithelial cells (Kunsmann et al., 2015; Bielaszewska et al., 2018), which may also have pathogenetic implications since proinflammatory cytokines play multiple roles in the pathogenesis of HUS (Zoja et al., 2010; Karpman et al., 2017). A deeper analysis of OMV-mediated IL-8 production demonstrated that flagellin and LPS are the key IL-8-inducing components of EHEC OMVs, and that flagellin-mediated signaling via TLR5, and LPS-mediated signaling via TLR4/MD-2 complex, followed by activation of the nuclear factor NF-κB, are the major pathways underlying IL-8 production (Kunsmann et al., 2015; Bielaszewska et al., 2018). The identification of EHEC OMVs as carriers for major EHEC virulence factors and powerful tools for their intracellular delivery and endothelial injury, combined with the proinflammatory and immunomodulatory activities of OMVs, allow to consider these nanostructures as novel virulence tools of EHEC which may play roles in the pathogenesis of EHEC-mediated diseases, in particular of HUS. This is further supported by a significantly increased EHEC OMV production under simulated human intestinal conditions and in the human intestine (Bauwens et al., 2017b). In contrast to ETEC OMVs where OMV-associated LT mediates the OMV interaction with the host cell via its interaction with GM_1_ (Horstman and Kuehn, 2002), the cellular uptake of EHEC OMVs is independent on OMV-associated virulence factors (Bielaszewska et al., 2017) and the cell receptor(s) for OMVs as well as their detailed internalization mechanism(s) remain unknown.

## Outlook

The roles of ETEC and EHEC OMVs as carriers for virulence factors and tools for their delivery into the host cells, together with OMV abilities to elicit immune responses against the major virulence proteins lead to attempts to exploit OMVs as vaccine candidates. OMVs are promising components of vaccines since they combine the antigen and adjuvant in a single formulation. A vaccine based on OMVs of a major EHEC serotype O157:H7 was found to protect against EHEC-mediated pathology in a mouse model and to be immunogenic in calves (Fingermann et al., 2018). These initial studies suggest that EHEC-derived OMVs have a potential for the formulation of both human and veterinary vaccines. However, further studies are needed to determine immunogenicity and protective efficacy of OMVs from other major EHEC serotypes associated with HUS (Karch et al., 2005), identify OMV components involved in the immune responses and mechanisms underlying OMV-elicited protective immunity.

The progress of development of OMV-based ETEC vaccines is more advanced than in EHEC. ETEC OMVs contain both confirmed and probable ETEC virulence factors (Table 1), which are highly immunogenic (Roy et al., 2010, 2011). Several studies with differently prepared OMVs from various strains demonstrated the immunogenic as well as protective effects of such vaccines in animal models (Roy et al., 2011; Leitner et al., 2015; Hays et al., 2018). Moreover, a combined OMV-based vaccine against ETEC and *V. cholerae* has been developed and shown to successfully protect against both pathogens (Leitner et al., 2015). However, a lot of additional work remains to be done before OMV-based vaccines against EHEC and ETEC infections can be used in clinical studies as it is already the case for vaccines against *Neisseria meningitidis* and *Neisseria gonorrhoeae* infections (Marsay et al., 2015; Petousis-Harris et al., 2017).

## Author Contributions

All authors listed have made a substantial, direct and intellectual contribution to the work, and approved it for publication.

### Conflict of Interest

The authors declare that the research was conducted in the absence of any commercial or financial relationships that could be construed as a potential conflict of interest.
